# Real-time PCR using *atp*E, conventional PCR targeting different regions of difference, and flow cytometry for confirmation of *Mycobacterium bovis* in buffaloes and cattle from the Delta area of Egypt

**DOI:** 10.1186/s12866-022-02568-0

**Published:** 2022-06-11

**Authors:** Mohamed Sabry Abd Elraheam Elsayed, Ahmed Salah, Ahmed Abd Elbadee, Tamer Roshdy

**Affiliations:** 1 Department of Bacteriology, Mycology, and Immunology, Faculty of Veterinary Medicine, University of Sadat City, Sadat City, Menoufia 32897 Egypt; 2 Department of Molecular Biology, Genetic Engineering, and Biotechnology Research Institute, University of Sadat City, Sadat City, Menoufia Egypt; 3 Animal Biotechnology Department, Genetic Engineering and Biotechnology Research Institute, University of Sadat City, Sadat City, Menoufia Egypt

**Keywords:** Cattle and buffalo, Conventional PCR, Flow cytometry, *Mycobacterium bovis*, Real-time PCR *atp*E

## Abstract

**Background:**

*Mycobacterium bovis* notoriously causes detrimental infections in bovines and humans. In this study, 1500 buffaloes and 2200 cattle were tested by single intradermal comparative cervical tuberculin test and compared with the detection rates of *M. bovis* isolation, real-time and simplex PCR, and flow Cytometry.

**Results:**

The tuberculin test is the reference test in Egypt, the positive rate was 54/3700 (1.5%) composed of 18/1500 (1.2%) buffaloes and 36/2200 (1.6%) cattle which were mandatorily slaughtered under the Egyptian legislation, after postmortem examination the non-visible-lesion proportion was 39/54 (72.2%) which surpassed the visible-lesion rate 15/54 (27.8%) with (*p* < 0.0001). The samples from each case were pooled into one sample representing the case, and the isolation rate of *M. bovis* was 25/54 (46.3%). Real-time PCR using *atp*E was positive for mycobacteria on the genus level in 18/18 (100%) and 5/5 (100%) of tissue samples and isolates, respectively; simplex PCR detected *M. bovis* in 44/54 (81.5%) and 25/25 (100%) of tissue samples and isolates, respectively. Flow Cytometry evaluation of the CD4^+^, CD8^+^, WC1^+^δγ, and CD2^+^ cell phenotypes showed increased counts in the tuberculin-positive cases compared with negative cases (*p* < 0.0001), and these phenotypes in the tuberculin-positive cases increased after antigen stimulation than in the negative cases (*p* < 0.0001). Detection rates of PCR techniques and flow Cytometry exceeded that of bacterial isolation (*p* < 0.0001) and exhibited a strong correlation.

**Conclusions:**

The skin test suffers from interference from non-tuberculous mycobacteria able to cause false-positive reactions in cattle and other species. Real-time PCR using *atp*E, conventional PCR targeting RDs, and flow Cytometry are rapid and accurate methods that correlate with the isolation and can be promising for detection and confirmation of infected live and slaughtered cases.

## Background

*Mycobacterium bovis* is a serious pathogen that causes chronic bovine tuberculosis (TB). It is a member of the *M. tuberculosis* complex (MTBC) which includes *M. tuberculosis*, *M. canettii*, *M. africanum*, *M. pinnipedii*, *M. microti*, *M. caprae*, and *M. mungi* which express a close genetic relationship and cause widely spread human and animal infections [1]. During the year 2016, zoonotic tuberculosis reached a rate of 147,000, with global deaths of 12,500 from *M. bovis* mainly in Africa [2]. Furthermore, since 1920, the prevalence of bovine tuberculosis in cattle and buffalo has ranged from 2 to 9% in Egypt [3]. During the year 1985, El-Olemy et al. [4], proved that 0.3% of 1003 cattle and 1.1% of 180 buffaloes were tuberculin-positive at Menoufia governorate. During the year 1980, bovine tuberculosis (bTB) in cattle and Egyptian buffaloes ranged between 6.9 and 26.2% then was reduced to 2.6% during 1990 [5]. Furthermore, Ramadan et al. [6], had confirmed that 1% of 3347 cattle were tuberculin-positive in 2011. Based on the official bTB database declared by the Egyptian GOVs, the infection rate had declined from 0.23 to 0.02 in 2009 to 2012 and then increased in 2013 as it reached 0.082 among Egyptian cattle [7]. During the period from 2015 to 2018, bTB represented 1.67% in 16 dairy herds in mid-Delta, Alexandria Road, and Upper Egypt districts [8]. The tuberculin-positive cases reached 1.31% in different Egyptian localities such as Alexandria, El-Behira, El-Gharbia, Kafr El-Sheikh, and Menoufia governorate within 2 years [9]. Furthermore, the tuberculous lesions in cattle and buffalo carcasses represented 0.7 and 0.45%, respectively from El-Mahalla El-Kubra abattoir, Mid-Delta of Egypt at 2016 [10]. In April 2019, a prevalence of 3.5% tuberculin-positive cases was declared by Elsayed and Amer [11], from cattle and Egyptian water buffaloes in Menoufia, Sharkia, Gharbia, Dakahlia, Elbuhaira, and Cairo. During August 2019, a prevalence of 3.43% was confirmed in 27 dairy herds from; Alexandria, Beheira, Daqahlia, Gharbia, Kafr EL Sheikh, Menoufia, and Sharkia [12]. With increased noticeable records, 16.4% of dairy cows were tuberculin-positive during 2020 from Al Dakahlia, Al Gharbia, Al Ismailia, Al Beheira, and Alexandria [13]. The tested Egyptian water buffalo species were distributed in the Nile Delta and Nile Valley, they were Baladi or called with other local names as; Beheri, Minufi, and Saidi. The Egyptian buffalo are either Riverine type which is colored black with long curled horns or Swamp type which color could be dark grey, black, black and white, or even all white with long gently curved horns [14].

Understanding the MTBC virulence factors is critical for the developing of novel vaccines and treatments to aid in the management of the disease as the world moves closer to becoming tuberculosis-free [15]. The cell envelope contains carbohydrates, lipids, and proteins covering the cell wall that interact with host cells [16]. The peptidoglycan, arabinogalactan, and mycolic acid in the cell wall are important in granuloma formation. The *M. bovis* outer membrane that contains mycolic acids and a capsule-like coat of polysaccharide and protein, confers a barrier to antibiotics, protect from immune system, and are necessary for survivability and pathogenesis [17]. The cord factor, a glycolipid defined as trehalose-6,6′ dimycolate, which depicts bacilli oriented in parallel form prevents leukocyte migration and is leukotoxic, causing swelling and disruption of liver mitochondria, disintegration of the rough endoplasmic reticulum, and ribosome detachment in liver cells. *M. bovis*’ host range and tissue tropism are influenced by the absence of sulfolipids and sulfatides from the cell wall. Mannosylated lipoarabinomannan (ManLAM), a prominent glycolipid in *M. bovis* is a ligand for interactions with dendritic and macrophage cells, allowing it to enter phagocytes via mannose receptors. LAM is a potent scavenger for reactive oxygen and nitrogen intermediates, which halt phagosome formation. Alkyl-hydroperoxidases protect *M. bovis* from reactive nitrogen intermediates. Antigen 85 catalyses the production of mono- and di-mycolyltrehalose, binds to fibronectin, prevents phagocytosis, and involved in cell-mediated immune response. In macrophages, the exported repetitive protein P36 is involved in intracellular replication. The heparin-binding haemagglutinin is implicated in the extrapulmonary spread. The mammalian cell entry proteins (Mce) are involved in invasion of phagocytic and epithelial cells. In *M. tuberculosis*, four operons each encode 5–6 proteins, but the *mce*3 operon is absent in *M. bovis*.

*M. bovis* phospholipases (A and B) have a cytotoxic effect on macrophages by hydrolyzing cell lipids. The oxidases defend *M. bovis* from reactive oxygen and nitrogen species in macrophages, allowing it to spread and persist more effectively. The proline-glutamate and proline-proline-glutamate proteins are essential for antigenic diversity, intracellular survival, humoral and cell-mediated responses. Superoxide dismutases, both iron-manganese-dependent (Sod A) and copper-zinc-dependent (Sod C) protect *M. bovis* from activated macrophages’ oxidative burst and reactive oxygen intermediates [18]. Virulent *M. bovis* is secreting large amounts of MPB70 and MPB83 it’s a membrane-bound homolog which have immunomodulatory functions as toll-like receptor (TLR)1/2 agonists and can induce tumor necrosis factor (TNF)-α, interleukin (IL)-6, and IL-12p40 [19–21].

Primers and probes targeting the *atp*E gene have previously shown accurate quantification and precise characterization of *Mycobacterium* sp. in environmental and clinical samples. This target expresses high sensitivity and specificity as it offers the merit of a gene present in a single copy in the genome [22], which is beneficial in detecting mycobacterial agents on the genus level. Regions of difference (RDs)-based genotyping is vital for unraveling the genetic diversity of the closely related MTBC members, and simplex or multiplex conventional PCR techniques have been designed using primers targeting RDs (RD1, 1mic, 2seal, 4, 9, and 12) to differentiate between these members [23]. Flow Cytometry is a useful tool for the detection and confirmation of different types of mycobacterial infections in animals. Many studies have used this technique to focus on the protective function of CD4^+^, CD8^+^, gamma Delta (γδ) T, and CD2^+^ cells. During infection with pathogenic mycobacteria, these cells exhibit changes in number and proportions that could be of diagnostic value [24]. The cell-mediated immune response is crucial to control mycobacterium infection. Dendritic cells (DCs), macrophages, neutrophils, natural killer (NK), γδ T cells, and their secreted cytokines play protective roles against such infections [25, 26].

The present work aimed to determine the prevalence of tuberculin-positive buffaloes and cattle from some governorates of the Delta area of Egypt. And to detect cases with visible and non-visible-lesions after postmortem examination. To isolate *M. bovis* on a specific medium and confirm it with real-time PCR using *atp*E primer/probe in cooperation with conventional PCR using primers targeting regions of difference. To make flow Cytometry evaluation of CD4^+^, CD8^+^, WC1^+^δγ, and CD2^+^ cell phenotypes for elucidating their counts in infected cases. To address the correlation, sensitivity, and specificity of the implemented research methods.

## Materials and methods

### Tuberculin testing

**Fig. 1:**
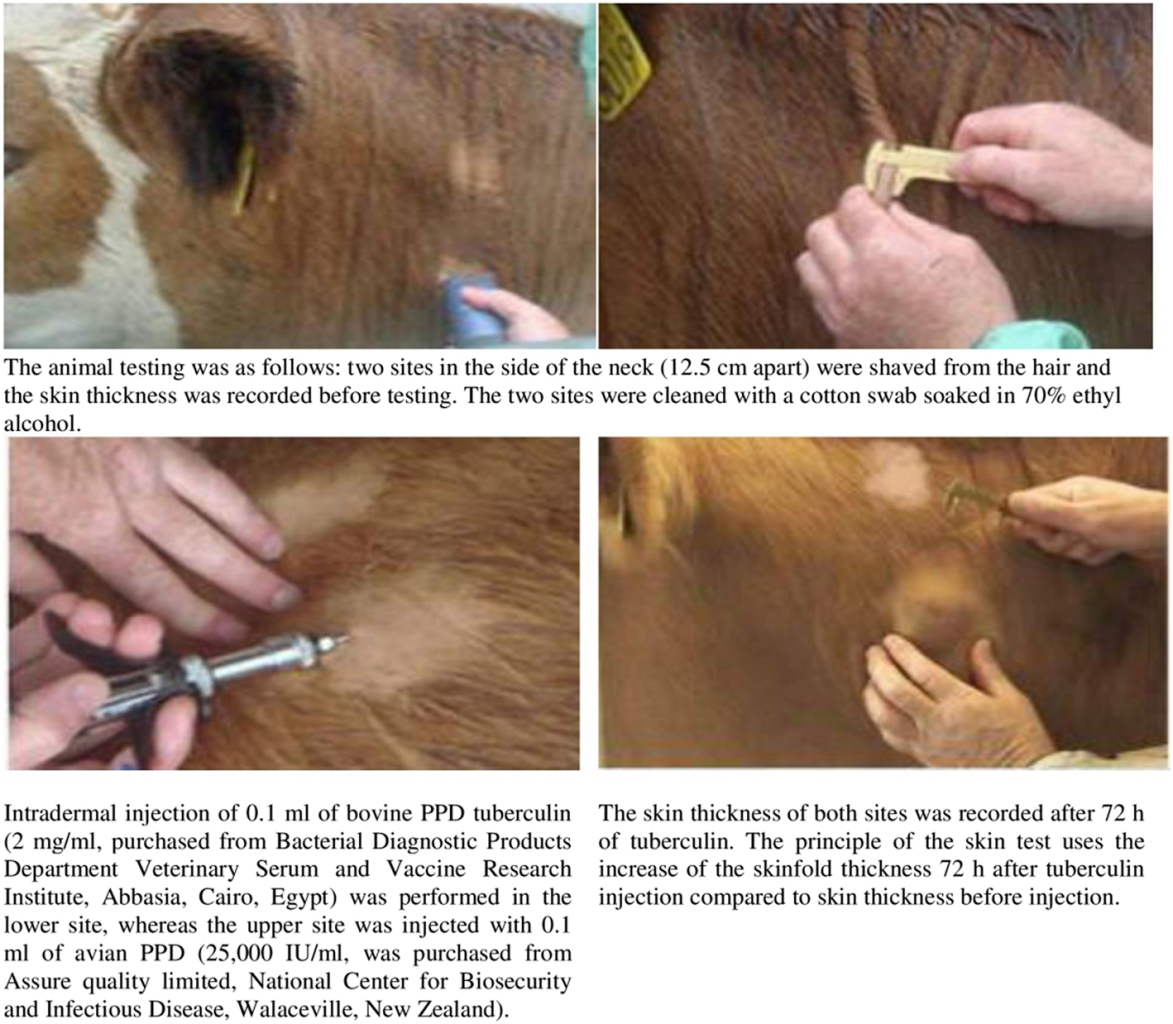
Steps of animal testing using single intradermal comparative cervical tuberculin

A total of 3700 animals (1500 native breed buffaloes and 2200 Holstein Friesian cattle) from Menoufia, Gharbia, Dakahlia, and El-Buhaira Governorate in Egypt were tested by single intradermal comparative cervical tuberculin during 2016–2019. The steps of animal testing were performed as illustrated in (Fig. 1). The interpretation of the test, is usually considered positive if the skin thickness at the bovine site is > 4 mm greater than the reaction at the avian injection site. Moreover, it is considered inconclusive when the increase in skin thickness at the bovine injection site is greater than the avian reaction with a difference < 4 mm. And it is considered negative if the increase in skin thickness at the bovine site of injection is less than or equal to the increase in the skin reaction at the avian site of injection [27]. The tuberculin negative cases with their previously registered ear tag numbers were retested again in the other neck side after 2 months.

This study, conducted in compliance with the ARRIVE guidelines for the care of the vertebrate animals implemented in training, testing, and research. The routine meat inspection in slaughterhouses was performed by following the Egyptian Law number 517 in 1986. The ethics committee of the faculty of veterinary medicine university of Sadat City Egypt, has approved the study for animal involvement in this study [The institutional animal care and use committee (IACUC)], the committee has also approved steps and protocol of this study with allotted number of 220/2018.

### Sampling and cultivation

#### Collection of samples

Blood samples were collected from tuberculin-positive cases (four buffalo and four cattle) and tuberculin-negative animals (four buffalo and four cattle). A volume of 37 ml blood was collected and mixed with a 12.5 ml acid citrate dextrose anticoagulant for separation of lymphocytes. All animals with positive reactions to the tuberculin test were slaughtered (according to Egyptian law) and exposed to postmortem examination; these animals were over 2 years of age. The lymph nodes; mandibular, tonsils, and the retropharyngeal of both sides, cranial and caudal mediastinal, left and right bronchial, hepatic, and mesenteric lymph nodes were examined and collected. Organ samples from the lungs, liver, spleen, and intestines were collected as well. These samples were sliced finely and examined grossly for lesions, and animals were classified as visible-lesion or non-visible-lesion. For the cultivation and isolation of *Mycobacterium bovis*, samples from each case were pooled to one sample representing the case and were subjected to decontamination and concentration using Petroff’s method [28].

#### Cultivation and identification

Each sample was cultivated onto four Lowenstein–Jensen (LJ) medium tubes (purchased from Thermo Fisher Scientific, Waltham, MA, USA): two tubes with sodium pyruvate (purchased from Thermo Fisher Scientific, Waltham, MA, USA) and the remaining two tubes with glycerol purchased from (ABID AG, Austria). One tube with pyruvate and one with glycerol were incubated in the light, and the others were incubated in the dark at 37 °C with 5% CO2. The isolates were identified after recording the rate of growth and microscopic detection of acid-fast bacilli using Ziehl–Neelsen staining [9, 29].

#### DNA extraction and molecular confirmation of different samples and isolates

DNA extraction from the lymph nodes and organ tissues was performed by QIAamp DNA extraction Miniprep Kit purchased from (Qiagen, Germany), according to the instructions of the manufacturer, especially for Gram-positive bacteria. Obtained isolates were killed by utilization of a chloroform: methanol 2:1 mixture purchased from (CLN GmbH, Germany). In a 1.5 ml Eppendorf tubes, a few colonies were selected and 200 μl Tris EDTA (TE) was added, then the tubes were subjected to overnight freezing. A volume of 200 μl of freshly prepared chloroform: methanol mixture was added to the tubes after thawing them. The tubes were subjected to centrifugation at 2000×g for 20 min with decanting of the supernatant. Subsequent centrifugation of the tubes was performed at 6000×g for 5 min followed by the discarding of the supernatant. Finally, the tubes were kept open to air dry for 1 h and then frozen until the beginning of DNA extraction. A combination of chemical, physical, and enzymatic procedures was performed for DNA extraction [30, 31].

#### Molecular confirmation of the *M. bovis*

##### Real-time PCR using *atp*E primer/probe

Primers and probe (Table 1) were purchased from Applied Biosystems, (Foster City, Calif., USA). Concentrations of primers were 900 nM for each, and that of the probe was 200 nM. The total reaction volume of PCR was 10 μL, including 5 μL of 2× LightCycler 480 Probes Master (ready to use hot start PCR mix), composed of dNTP mix (with dUTP instead of dTTP), reaction buffer, FastStart Taq DNA polymerase, and 6.4 mM MgCl_2_, forward and reverse primers with a concentration of 0.5 mM), FAM-labeled probe with a concentration of 0.2 mM, dimethyl sulfoxide 4% purchased from (Takara Bio Inc., Otsu, Shiga, Japan), and 1 μL of template DNA, and the volume was completed with 3.5 μL of RNAse-free dH_2_O [22].

**Table 1:** The primers and probes of the targeted genes in this study

Type of PCR	Target gene	Primers and probe sequences (5′-3′)	Amplicon size (bp)	Annealing temperature (°C)	Melting temperature (°C)	Accession number	Reference
Real-time	*atp*E	F 5′-CGGGCCGGATCGGGA-3′ R 5′-CGAAGACGAACAGCCAT-3′ P FAM 5′-ACGTGATGAAGAACGGGT AA-3′	182	60	63.7 57.1 58.9	CP023630.1	[22]
Conventional^a^	RD1	5′-AAGCGGTTGCCGCCGACCGACC-3′ 5′-CTGGCTATATTCCTGGGCCCGG-3′ 5′-GAGGCGATCTGGCGGTTTGGGG-3′	146	62	60.3 63.4 66	CP009186.1	[32]
	RD4	5′-ATGTGCGAGCTGAGCGATG-3′ 5′-TGTACTATGCTGACCCATGCG-3′ 5′-AAAGGAGCACCATCGTCCAC-3′	172		67.5 66.7 67.1	CP009186.1	
	RD9	5′-CAAGTTGCCGTTTCGAGCC-3′ 5′-CAATGTTTGTTGCGCTGC-3′ 5′-GCTACCCTCGACCAAGTGTT-3′	235		66.6 63.9 66.5	CP009186.1	
	RD12	5′-GGGAGCCCAGCATTTACCTC-3′ 5′-GTGTTGCGGGAATTACTCGG-3′ 5′-AGCAGGAGCGGTTGGATATTC-3′	369		67 65.7 66.9	CP009186.1	

^a^RD-based detection of *M. bovis* as follows; RD1 present (146 bp), RD4 absent (268 bp), RD9 absent (108 bp), and RD12 absent (306 bp)

The real-time PCR technique was achieved by comparison against standard curves of control DNA with tenfold concentrations (100,000, 1000, 100, 10, 1, 0.1, and 0.01 ng/μL) extracted from *M. bovis* BCG (ATCC 19210), *M. tuberculosis* H37RV, and internal positive *M. bovis* isolate as positive controls and *E. coli* (ATCC 11775) as a negative control. Control DNA samples were quantified using the Qubit dsDNA HS high sensitivity assay kit, purchased from (Thermo Fisher Scientific, USA). DNA quantification and the real-time PCR were performed at the Department of Molecular Biology, Genetic Engineering and Biotechnology Research Institute, University of Sadat City, Sadat City, Menoufia, Egypt. The utilized machine was the LightCycler 480InstrumentII-Roche Life Science with its software version LCS480 1.5.0.39.

##### Conventional PCR based on the presence or absence of genomic regions of difference

All the utilized primers (Table 1) were purchased from (Takara, Japan). The protocol and conditions of PCR were achieved as follows for a total PCR volume of 12.5 μL: 1 μL of the sample DNA, 2 μL of 25 mM MgCl_2_, 4 μL of 10 mM dNTPs, 5 μL of the high G-C buffer, 2.5 μL of the 10x buffer, 0.5 μL of each primer (50 pmol/μL), and 0.125 μL of the HotStar Taq DNA polymerase purchased from (Biometra, Germany), and the volume was completed to 12.5 μL with RNAse-free H_2_O. Amplification was begun with incubation at 95 °C for 5 min, and then followed by 45 cycles at 94 °C for 1 min, 62 °C for 1 min, and 72 °C for 1 min. Samples were kept at 72 °C for 10 min [32]. Both the *M. bovis* (ATCC35734D-2) and internal positive *M. bovis* isolate were used as positive controls, and *E. coli* (ATCC 11775) was used as a negative control.

The possible target regions and accession numbers have been identified using the available web-based software at https://blast.ncbi.nlm.nih.gov/Blast.cgi?PROGRAM=blastn&PAGE_TYPE=BlastSearch&LINK_LOC=blasthome. Thes methods used to calculate the melting temperature have also been confirmed with the utilization of OligoAnalyzer 3.1 (Integrated DNA Technologies, Inc.: https://eu.idtdna.com/calc/analyzer). Conventional PCR was performed at the laboratory of the Department of Bacteriology, Mycology, and Immunology, Faculty of Veterinary Medicine, University of Sadat City, Egypt with a SimpliAmp Thermal Cycler-Thermo Fisher Scientific. The QIAxcel machine and specified software (screenGel software 1.6 standard upgrade) for data analysis were used for visualization of the PCR amplification products.

### Flow cytometry

#### Animal grouping for blood sampling

The first group contained eight naturally infected cases with *M. bovis* (four buffalo and four cattle) that were tuberculin-positive and confirmed by culture, real-time PCR using *atp*E, and RD-based conventional PCR. The second group (control group) contained eight (four buffalo and four cattle) that were tuberculin-negative.

#### Direct immunofluorescence staining of peripheral blood mononuclear cell surface epitopes

Immunofluorescence staining was performed according to the Bio-Rad AbD Serotec protocol. Briefly, a volume of 2 ml of freshly prepared RBCs lysis buffer (erythrolyse) was added to a 50 ml falcon tube containing 48 ml blood, and the tubes were incubated at room temperature for 10 min. The tubes were centrifuged at 300–400 xg for 5 min at room temperature, and the supernatant was discarded. The tubes were washed with a volume of 2 ml phosphate buffer saline/bovine serum albumin (PBS/BSA) kept at room temperature and centrifuged at 300–400 g for 5 min with subsequent discarding of the supernatant. The cell count was adjusted using cold (4 °C) PBS/BSA buffer to 1 × 10^7^ cells/ml. A volume of 100 μL of the cell suspension was dispensed from each sample into four cryotubes. Monoclonal antibodies (Table 2), and purchased from (Bio-Rad AbD Serotec, UK) were prepared at the vendor-recommended dilution, and a volume of 100 μL from the four types was added to each tube. Tubes were mixed and incubated at 4 °C for 30 min in the dark. Tubes were washed with 2 ml cold (4 °C) PBS/BSA, and the tubes were centrifuged at 300-400×g for 5 min at 4 °C; the supernatant was discarded. The cell pellet was re-suspended with 200 μL of 0.5% paraformaldehyde diluted in PBS [24].

**Table 2:** Utilized monoclonal antibodies

Product code	Ig isotype	Specificity
MCA1653F	IgG2a	CD4
MCA837F	IgG2a	CD8 CD8 alpha
MCA838G	IgG2a	WC1+ δγ TCR1 chain
MCA833F	IgG1	CD2

A Becton Dickinson FACS Caliber flow Cytometer equipped with argon and red lasers was used for the analysis of the cells. This machine was controlled by a Macintosh 5 computer, with Cell Quest software (Becton Dickinson Immunochemistry systems, San Jose, CA, USA) at the Flow Cytometry Unit, Department of Clinical Pathology, Faculty of Medicine, Menoufia University, Egypt. FCS expresses software (De Novo Software, Thornton, Ontario, Canada, version 7.08.0018) used to analyze the data.

#### Staining of cell surface epitopes after stimulation with different antigens

The wells of the microtiter plates (96-well round-bottom) purchased from (Falcon; Becton-Dickinson, Lincoln Park, NJ) were seeded with 5 × 10^5^ peripheral blood mononuclear cells (PBMCs) in a total volume of 200 μL per well from separated lymphocytes after the lysis of red blood cells of each blood sample. RPMI 1640 medium purchased from (Merck KGaA, Egypt) was used and supplemented with 2 mM L-glutamine, 25 mM HEPES buffer, 100 U/ml penicillin, 100 μg/ml streptomycin, 1% nonessential amino acids (Merck KGaA, Egypt), 2% essential amino acids (Merck KGaA, Egypt), 1% sodium pyruvate (Merck KGaA, Egypt), 50 μM 2-mercaptoethanol, and 10% (vol/vol) fetal bovine serum. The PBMCs from each case was dispensed into four Wells: the first contained medium-plus *M. bovis* PPD (5 μg/ml) purchased from (Bacterial Diagnostic Products Department Veterinary Serum and Vaccine Research Institute, Abbasia, Cairo, Egypt), the second contained medium-plus ESAT-6 (10 μg/ml)/CFP10 (10 μg/ml) purchased from (MyBioSource, USA), the third contained medium-plus MBP70 (10 μg/ml) purchased from (MyBioSource, USA), and the fourth contained cells dispensed in RPMI medium. Wells containing medium alone were used as blanks. The plates were incubated for 3 days at 37 °C in 5% CO2. Finally, the cells from each case either un-stimulated or stimulated with different antigens were labeled with the four monoclonal antibodies and fixed like the direct labeling method [33].

#### Data analysis

The statistical analysis system software package SAS for Windows, version 8 (SAS Institute, Cary, NC, USA) was implemented for data analysis. Significance of the difference between visible-lesion cases, non-visible-lesion cases, results of isolation, real-time PCR, conventional PCR, and flow Cytometry was elucidated using the free online posthoc Tukey’s honestly significant difference (HSD) calculator available at http://astatsa.com/OneWay_Anovawith_TukeyHSD/. The results of the real-time PCR were collected after the analysis of the LightCycler 480 software version LCS480 1.5.0.39 for the determination of mean crossing point (CP), standard crossing point (Std Cp), mean concentration (conc), and standard concentration (Stdconc) to elucidate the positive samples. The standard errors of the means of different cell phenotypes evaluated by flow Cytometry were detected using the free online calculator available at https://goodcalculators.com/standard-error-calculator/. Determination of correlation coefficients of isolation, real-time PCR, conventional PCR, and flow Cytometry was detected using the free online calculator available at http://www.learningaboutelectronics.com/Articles/Rsquaredcalculator.php#answer. The sensitivity and specificity for all used research techniques were detected using the free online calculator available at https://www.medcalc.org/calc/diagnostic_test.php_ using the result of isolation which was considered as the gold standard.

## Results

### Tuberculin testing and postmortem examination

A total of 1500 buffaloes were tested with the comparative intradermal tuberculin test. The overall prevalence of tuberculin-positive cases was 18/1500 (1.2%), which contained 18/1500 (1.2%) and 36/2200 (1.6%) tuberculin-positive cases for buffaloes and cattle, respectively. Tuberculin-positive buffaloes were slaughtered and subjected to postmortem examination. The proportion of non-visible-lesion cases was 17/18 (94.4%), higher than that of visible-lesion cases 1/18 (5.6%) and there was a significant difference between them (*p* < 0.0001). A total of 2200 cattle was tested, and the prevalence of tuberculin-positive cases was 36/2200 (1.6%) slightly higher than that of buffaloes. The sensitivity and specificity of the tuberculin test for buffalo was 27.78 and 100% respectively, while the sensitivity and specificity of tuberculin test for cattle were 55.56 and 100% respectively. Tuberculin-positive cattle were slaughtered and subjected to thorough meat inspection. The rate of non-visible cases was 22/36 (61.1%), significantly higher than that of the visible-lesion expressing cases 14/36 (38.9%) with (*p* < 0.0001). The overall prevalence of tuberculin-positive cases was 54/3700 (1.5%), the proportion of non-visible cases was 39/54 (72.2%), and the rate of cases expressing visible-lesions was 15/54 (27.8%) with a confirmed significant difference (*p* < 0.0001). The cases that expressed visible lesions gave skin thickness more than 5 mm in both buffaloes and cattle. The buffalo case 1/18 (100%) showed visible-lesion expressing the lesions in the lung and pulmonary lymph nodes. Moreover, the cattle visible lesion cases represented 14/36 (38.9%), from the 8/14 (57.1%) showed lesions in the lung and pulmonary lymph nodes while 6/14 (42.9%) gave generalized lesions that appeared in lung and pulmonary lymph nodes with mesenteric lymph nodes of the same case. In addition to that, the cases that expressed non-visible lesions gave skin thickness more than 4 mm and lower than 5 mm and represented 17/18 (94.4%) and 22/36 (61.1%) of buffaloes and cattle, respectively with slight congestion in their L. nods (Table 3). The tuberculin test was repeated 2 months later for buffaloes and cattle cases that previously produced negative results in the other neck side and confirmed their negative status to avoid the false-negative results.

**Table 3:** Detailed descriptions of postmortem examination, *M. bovis* isolation, and results of single intradermal comparative cervical tuberculin test

Postmortem examination	Site of lesion	Number				Results of single intradermal comparative cervical tuberculin test
		Buffaloes	*M. bovis* isolation	Cattle	*M. bovis* isolation	
1. Visible lesion (VL)	1.1localized					Skin thickness > 5 mm
	a. Head L.nods					
	b. Lung and pulmonary L.nods	1/18 (5.6%)	1/18 (5.6%)	8/36 (22.2%)	8/36 (22.2%)	
	c. Mesenteric L.nods					
	1.2 Generalized^a^			6/36 (16.7%)	6/14 (16.7%)	
Total		1/18 (5.6%)		14/36 (38.9%)	14/36 (38.9%)	
2. Non-visible lesion (NVL)	Congested L.nods	17/18 (94.4%)	4/18 (22.2%)	22/36 (61.1%)	6/36 (16.7%)	4 mm < Skin thickness < 5 mm
Total		18	5/18 (27.8%)	36	20/36 (55.6%)	

^a^Generalized; Lesions found in lung and pulmonary L.nods with mesenteric L.nods of the same case

### Isolation and molecular confirmation

**Fig. 2:**
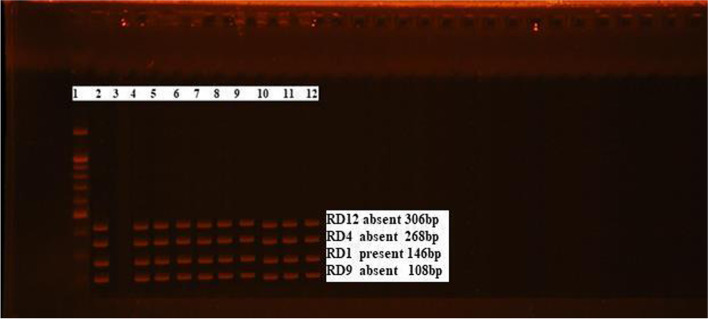
Electrophoretic pattern of positive *M. bovis* isolates using QIAxcel machine. *M. bovis* characterized by RD1 present at (146 bp), RD4 absent at (268 bp), RD9 absent at (108 bp), and RD12 absent at (306 bp)

**Fig. 3:**
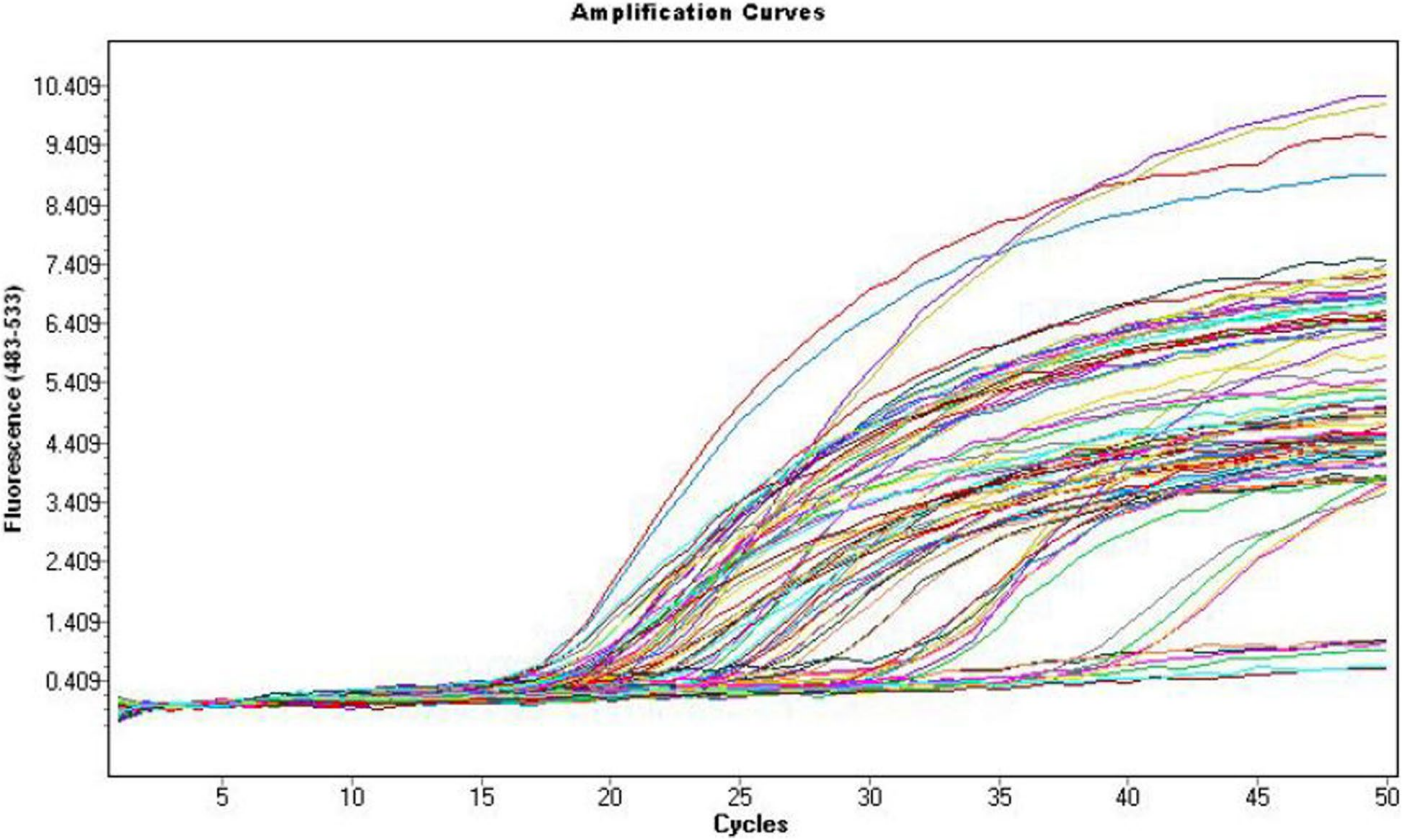
Amplification curve of real-time PCR assay using *atp*E primer/probe. The fluorescence increase wasn’t detectable until cycle 20 as enough amplified product was accumulated to yield a detectable fluorescence signal using control DNA with concentrations of (100,000, 1000, 100, 10, 1, 0.1, and 0.01 ng/μL). The crossing points and concentrations were automatically calculated by the real-time PCR software (LightCycler 480 Roche)

The results of isolation confirmed that 5/18 (27.8%) buffaloes and 20/36 (55.6%) cattle were positive for *M. bovis* isolation, with a significant difference between them (*p* < 0.0001), with an overall rate of the isolated *M. bovis* was 25/54 (46.3%), and all cases showing visible-lesions were positive for isolation while no isolates were obtained from the non-visible lesion cases. Real-time PCR using *atp*E primer/probe confirmed the existence of mycobacteria on the genus level in all the DNA extracted from the tissue samples and isolates with rates of 54/54 (100%) and 25/25 (100%), respectively. These results confirmed a significant difference between real-time PCR and the results of isolation (*p* < 0.0001). The genomic RD-based simplex PCR detected *M. bovis* at a rate of 14/18 (77.8%) and 30/36 (83.3%) from infected buffalo and cattle cases, respectively, with an overall rate of 44/54 (81.5%) from tissue samples and 25/25 (100%) from isolates, which was significantly different from the results of isolation (*p* < 0.0001). The RD-based detection of *M. bovis* revealed that RD1 present (146 bp), RD4 absent (268 bp), RD9 absent (108 bp), and RD12 absent (306 bp). The overall detection rate of the real-time PCR targeting *atp*E gene expressed a significant difference from the isolation and the conventional PCR on tissue samples with (*p* < 0.0001), (Table 4, Figs. 2 and 3). The real-time PCR uses primer/probe for *atp*E that detects mycobacterial agents on the genus level while the conventional PCR using primers targeting the regions of difference that identifies *M. bovis* on the species level.

**Table 4:** Results of tuberculin Test, postmortem examination, isolation, real-time PCR using *atp*E, and genomic RD-based PCR

Type of animal	Total no. of tested animals	Comparative intradermal tuberculin test	Sensitivity of tuberculin*	Specificity of tuberculin*	Postmortem examination		Culture on LJ with pyruvate**	Real-time PCR using *atp*E		RD based conventional PCR for confirmation of *M. bovis*	
					Visible-lesion	Non visible-lesion		Tissue	Culture	Tissue	Culture
Buffaloes	1500	18 (1.2%)	27.78%	100%	1/18 (5.6%)^a^	17/18 (94.4%)^b^	5/18 (27.8%)^a^	18/18 (100%)^b^	5/5 (100%)^b^	14/18 (77.8%)^b^	5/5 (100%)^b^
Cattle	2200	36 (1.6%)	55.56%	100%	14/36 (38.9%)^a^	36/22 (61.1%)^b^	20/36 (55.6%)^a^	36/36 (100%)^b^	20/20 (100%)^b^	30/36 (83.3%)^b^	20/20 (100%)^b^
Total	3700	54 (1.5%)			15/54 (27.8%)^a^	39/54 (72.2%)^b^	25/54 (46.3%) ^a^	54/54 (100%)^b^	25/25 (100%) ^b^	44/54 (81.5%) ^b^	25/25 (100%)^b^

*The sensitivity and specificity of the comparative intradermal tuberculin test were calculated in relation to isolation

**All the cases showing gross lesions were positive for isolation, while all the non-visible cases were negative for isolation

Superscript letters a and b within the same row represent results that are significantly different (*p* < 0.0001)

### Flow cytometry evaluation of CD4^+^, CD8^+^, WC1^+^δγ, and CD2^+^ cell phenotypes after direct labeling from infected cases and stimulated with specific mycobacterial antigens

Direct labeling of lymphocytes from tuberculin-positive and tuberculin-negative buffaloes demonstrated that counts of CD4^**+**^, CD8^**+**^, WC1^+^δγ, and CD2^+^ cell phenotypes reflected a significantly high induction in tuberculin-positive buffaloes when compared with tuberculin negative cases with (*p* < 0.0001), (Table 5). The same difference was observed for cattle (*p* < 0.0001) (Table 4). The numbers of the CD4^+^, CD8^+^, WC1^+^δγ, and CD2^+^ cell phenotypes of tuberculin-positive buffaloes and cattle were higher than that of tuberculin-negative counterparts (*p* < 0.0001) when these cells were isolated on the RPMI without any stimulating antigen (Table 6). The numbers of CD4^+^, CD8^+^, WC1^+^δγ, and CD2^+^ cell phenotypes from the tuberculin-positive buffaloes and cattle furthermore reflected increased lymphocyte activation with PPDB, ESAT-6/CFP10, and MBP70 antigens and significant difference arose when compared with the tuberculin-negative counterparts stimulated with the same antigens with (*p* < 0.0001), (Table 7).

**Table 5:** The results of direct readings at time zero (T0) before stimulation of eight buffaloes and eight cattle

Type and number of animals	Cell phenotypes			
	CD4^+^	CD8^+^	WC1^+^δγ	CD2^+^
Tuberculin negative buffaloes (4)	2255 ± 390^a^	2275 ± 390^a^	1900 ± 404^a^	2400 ± 115^a^
Tuberculin positive buffaloes (4)	4555 ± 1228^b^	2950 ± 202^b^	3800 ± 273^b^	2825 ± 558^b^
Confidence Intervals (95% CI)	[723.65, 3876.35]	[137.65, 1212.35]	[1303.45, 2496.55]	[− 272.04, 1122.04]
Tuberculin negative cattle (4)	1150 ± 28^a^	850 ± 144^a^	1950 ± 144^a^	1700 ± 404^a^
Tuberculin positive cattle (4)	3900 ± 692^b^	1350 ± 144^b^	2150 ± 663^b^	3400 ± 173^b^
Confidence Intervals (95% CI)	[1902.68, 3597.32]	[250.85,749.15]	[−630.06, 1030.06]	[1162.31, 2237.69]

These data expressed as Mean ± SE of the percentage readings to 10,000 peripheral blood mononuclear cells (PBMC)

Each value represents the mean of 4 cases. Superscript letters a and b within the same column to the relevant species representing cases that are significantly different (*p* < 0.0001)

**Table 6:** Results of different cell phenotypes of eight buffaloes and eight cattle on RPMI medium without antigen stimulation

Type and number of animals	Cell phenotypes			
	CD4^+^	CD8^+^	WC1^+^δγ	CD2^+^
Tuberculin negative buffaloes (4)	2350 ± 144^a^	2428 ± 962^a^	2145 ± 230^a^	3100 ± 257^a^
Tuberculin positive buffaloes (4)	3275 ± 1688^b^	2750 ± 202^b^	2450 **±** 228^b^	3995 ± 225^b^
Tuberculin negative cattle (4)	4300 ± 230^a^	4550 ± 28^a^	4350 ± 202^a^	3950 ± 548^a^
Tuberculin positive cattle (4)	4900 ± 461^b^	4980 ± 404^b^	4500 ± 346^b^	4390 ± 327^b^

These data expressed as Mean ± SE of the percentage readings to 10,000 peripheral blood mononuclear cells (PBMC)

Each value represents the mean of 4 cases. Superscript letters a and b within the same column to the relevant species representing cases that are significantly different (*p* < 0.0001)

**Table 7:** Readings of different cell phenotypes from eight buffaloes and eight cattle on RPMI medium with different antigens for cellular stimulation

The antigens used for stimulation	Type of animal	Tuberculintest	Cell phenotypes			
			CD4^+^	CD8^+^	WC1^+^δγ	CD2^+^
PPDB	Buffaloes	-ve	2233 ± 327^a^	2016 ± 394^a^	2000 ± 137^a^	2666.7 ± 153^a^
		+ve	3600 ± 255^b^	2950 ± 28^b^	2950 ± 375^b^	2950 ± 259^b^
MBP70		-ve	1833 ± 57^a^	4250 ± 86^a^	1300 ± 250^a^	2366 ± 288^a^
		+ve	2700 ± 404^b^	4500 ± 808^b^	2400 ± 115^b^	3300 ± 115^b^
ESAT6/CFP10		-ve	2900 ± 230^a^	2760 ± 115^a^	2500 ± 490^a^	2250 ± 317^a^
		**+**ve	3000 ± 404^b^	3450 ± 144^b^	2700 ± 173^b^	2800 ± 115^b^
PPDB	Cattle	-ve	6266.7 ± 296^a^	5300 ± 472^a^	6150 ± 683^a^	4300 ± 827^a^
		+ve	6666 ± 706^b^	6350 ± 541^b^	6550 ± 518^b^	4950 ± 715b
MBP70		-ve	4750 ± 747^a^	3900 ± 545^a^	4850 ± 1007^a^	4300 ± 626^a^
		+ve	718 ± 5550^b^	5916.7 ± 639^b^	5966.7 ± 455^b^	4650 ± 559^b^
ESAT6/CFP10		-ve	5950 ± 836^a^	4166.7 ± 492^a^	5033.3 ± 709^a^	4400 ± 89^a^
		+ve	6416.7 ± 874^b^	5866 ± 620^b^	6150 ± 413^b^	6300 ± 670^b^

These data expressed as Mean ± SE of the percentage readings to 10,000 peripheral blood mononuclear cells (PBMC)

Each value represents the mean of 8 cases. Superscript letters a and b within the same column to the relevant species representing cases that are significantly different (*p* < 0.0001)

### Determination of correlation, sensitivity, and specificity of different used research methods

Based on the results of these research methods, and correlation analysis, a strong correlation arose between the results of *M. bovis* isolation, real-time PCR, conventional PCR targeting RDs, and flow Cytometry with coefficients of 1 for each, respectively. The results of sensitivity and specificity of real-time PCR, and flow Cytometry were 100 and 100%, respectively, and for conventional PCR targeting different RDs, they were 81.48 and 100% when compared with *M. bovis* isolation as the gold standard. The results of sensitivity and specificity of *M. bovis* isolation were 46.30 and 100%, respectively, with decreased sensitivity than the other implemented methods (Table 8).

**Table 8:** Correlation, sensitivity, and specificity of different used research methods

Statistical parameters	Isolation	Real-time PCR	Conventional PCR targeting RDs	Flow Cytometry
Correlation coefficient	1	1	1	1
Sensitivity	46.30%	100%	81.48%	100%
Specificity	100%	100%	100%	100%

The correlation between isolation, real-time PCR, conventional PCR, and flow Cytometry. The sensitivity and specificity were calculated using isolation as a gold standard

## Discussion

*M. bovis* is a serious zoonotic threat; bovine tuberculosis has emerged as an ever-growing disease of animal populations causing severe economic losses in many countries [34]. We found a prevalence of 1.2 and 1.6% tuberculin-positive cases for buffaloes and cattle, respectively, with an overall rate of 1.5%. This is nearly similar to published reports from Egypt 2016 [9], and lower than the published data at the beginning of 2019 [11] and the mid of 2019 [35]. This fluctuation of records may be due to the different times of animal testing and the strict implementation of test and slaughter policy adopted by the General Authority for Veterinary Services for controlling mycobacterium infections in animals and potentially spreading to humans [3]. Despite its flaws, the tuberculin test (TST) is still the standard field test for screening African buffaloes for *M. bovis* infection, with results interpreted using cattle interpretation criteria. However, the TST has yet to be confirmed in African buffaloes, and calculating species-specific cut-off values could help enhance test results [36]. According to the current OIE standards, a bTB-positive test result for terrestrial species should have a 4 mm skinfold thickness increase at the bovine PPD injection site over the avian PPD site [27, 37] .

The postmortem examination of the slaughtered tuberculin-positive cases elucidated that the visible-lesion cases were lower than those of the non-visible lesion cases. This is contrary to records from Taiwan during 2013 [38], Egypt during 2016 [34], and 2019 [35]. The high rates of non-visible lesions may be attributable to the early stage of infection with *M. bovis*, as these animals during the early stage could react with cell-mediated immune response-based diagnostic tests such as the tuberculin test and the IFN-γ release assay with no appearance of lesions [39]. Moreover, the route of infection is considered one of the important factors controlling the development of the visible lesions, Serrano et al. [39]; found the absence of gross lesions in 60% of calves slaughtered 12 weeks after *M. bovis* oral challenge. *M. bovis* isolation yielded 46.3% samples that grew on LJ with sodium pyruvate, and all cases showing visible gross lesions were positive for isolation; this rate was similar to records from India during 2008 [40]. The lower isolation rate on specific media could be because most cases were non-visible as the burden of *M. bovis* was low in the lymph nodes and organ tissue and high in the blood, as opposed to the cases expressing visible-lesions which gave higher isolation due to the high burden of *M. bovis* in the lymph nodes and organ tissue than in the blood [41]. In addition, the increased isolation rate of cattle compared with the native breed of buffalo could be regarded to the breed of cattle, which was Holstein Friesian and not a native breed of Egypt; they were imported into the country and exposed to stressful rearing and environmental conditions. Moreover, the disease management procedures, uncontrolled cattle movement, and intensified husbandry systems are considered predisposing factors [42, 43].

The implementation of real-time PCR using *atp*E primer/probe proved that all the DNA from the tissue samples and isolates harbored mycobacteria. These results agree with the published records from Egypt [11]. The high detection results of the real-time PCR using *atp*E primer/probe were based on the ability of this real-time PCR technique to quantify very few mycobacterial DNA copies in the tissue samples and isolates [22, 44]. The *atp*E gene codes for ATP synthase subunit C in the genome of *M. tuberculosis* H37Rv (locus Rv1305); the encoded protein is a target of the new antimycobacterial diarylquinoline R2079100 that shows a bactericidal effect on mycobacteria [45, 46]. Furthermore, the in vitro results showed the specificity of the *atp*E gene for the detection of mycobacteria on the genus level. Comparative genomics with the entire DNA sequence of *M. tuberculosis* H37Rv has resulted in the demonstration of 16 genomic regions of difference [47]. The RD-based simplex PCR identified *M. bovis* in the infected buffalo and cattle cases with a frequency of 77.8 and 83.3%, respectively, at a maximum rate of 81.5% were substantially different from the findings of bacterial isolation. These results were higher than Elsayed and Amer [11], and confirmed the importance of the genotypic analysis of different RDs that elucidates the genetic diversity of the closely related MTBC members. The conventional PCR based on different RDs produced lower results when compared with real-time PCR using *atp*E, due to ability of real-time PCR using *atp*E to detect mycobacteria on the genus level while the conventional PCR could detect the *M. bovis* on the species level. These results confirm that the skin test is known to suffer from interference from non-tuberculous mycobacteria able to cause false-positive reactions in cattle and other species. The application of flow Cytometry has added progress in the field of tuberculosis diagnosis and understanding the underlying immunology [48]. The active immune responses to mycobacterium infections primarily depend on cell-mediated immune responses or Th1 reactions that include macrophages, dendritic cells, and adaptive T-cell responses. The cytokines that originate from antigen-specific T cells, with the key cytokine IFN-γ, regulate these responses [26]. The analysis of lymphocyte subsets in the circulation of animals experimentally infected with *M. bovis* showed the sequential role of the γδ, CD4^+^, and CD8^+^ T cells during infection. CD4^+^ T cells appear to be the main cell population in *M. bovis*-infected bovines, producing IFN-γ and leading to the activation of macrophage anti-mycobacterial capacity, with CD8^+^ T cells more involved in the infected cell lysis [49]. NK are the primary cells of the innate immune response to mycobacterium infection. These cells can make lysis of infected cells and secret immunomodulating cytokines such as IFN-γ without any prior sensitization. NK cells can help T cells polarize toward the Th1 phenotype and promote the maturation of DC [50]. Direct labeling of different cell phenotypes herein showed that CD2^+^, CD4^+^, CD8^+^, and WC1^+^δγ cells were higher in the tuberculin-positive than the tuberculin-negative buffalo and cattle. These results were confirmed by Pinheiro et al. [51], who elucidated the activation of CD4^+^, CD8^+^, and γδ T-cells during tuberculosis. In addition, the activation and expansion of CD2^+^ NK cells during the mycobacterial infection with the production of IFN-γ and perforin were confirmed by Junqueira-Kipnis et al. [52]. The counts of CD4^+^, CD8^+^, WC1^+^δγ, and CD2^+^ cell phenotypes from the tuberculin-positive buffaloes and cattle reflected increased cell phenotypes after stimulation with the used PPDB, ESAT-6/CFP10, and MBP70 antigens [24, 53, 54]. Furthermore, ESAT-6/CFP-10 imparted a stimulatory effect on these cell phenotypes [55–57]. The MBP70 a homolog of MPB83 also caused expansion of these cell phenotypes [58–61]. A systematic application of tuberculin screening and the culling of reactor cattle has been accomplished in many countries and resulted in the successful eradication of bovine tuberculosis [62]. There is a noticeable positive correlation between the *M. bovis* isolation, real-time PCR using *atp*E primer/probe on the genus level in cooperation with conventional PCR targeting different (RDs) on the species level, and flow Cytometry. It was clear that the sensitivity and specificity of the real-time PCR using *atp*E primer/probe, conventional PCR targeting different (RDs), and flow Cytometry surpassed that of tuberculin test and isolation on specific media. The interpretation of this result is that the real-time PCR using *atp*E primer/probe on the genus level in cooperation with conventional PCR targeting different (RDs) on the species level was performed using specific targets [22], and flow Cytometry measured specific cell phenotypes incorporated in the response to *M. bovis* and utilized specific mycobacterial antigens for stimulation of different cells [24, 53, 54]. *M. bovis* isolation, however, is affected by many factors such as bacterial load in the sample [42], decontamination method used, and type of media [63].

## Conclusion

From the results, although the results of the tuberculin test indicate the decreasing frequency in buffaloes and cattle. The results of tuberculin test confirmed flaws when compared with that of *M. bovis* isolation, real-time PCR using *atp*E primer/probe on the genus level in cooperation with conventional PCR targeting different regions of difference on the species level, and flow Cytometry and call for the implementation of highly sensitive and specific methods. Although isolation is the gold standard for confirmation of *M. bovis* infection, the combination of real-time PCR using *atp*E primer/probe and conventional PCR targeting different regions of difference could be beneficial for confirming the existence of the microorganism in tissue samples and culture. The flow Cytometry evaluation of CD4^**+**^, CD8^**+**^, WC1^+^δγ, and CD2^+^ cell phenotypes from infected cases reflects significantly high counts especially when stimulated with PPDB, ESAT-6/CFP10, and MBP70 antigens. These methods conferred high sensitivity and specificity when compared with mycobacterial isolation, an extremely time-consuming method. Furthermore, these techniques could differentiate infected cases from non-infected ones. Hence, these research techniques will help in the improvement of tuberculosis control strategies and judgment of the most effective treatment regimen.

### Study limitations

The General Authority for Veterinary Services and the Egyptian Ministry of Agriculture strictly implement the test and slaughter policy adopted for controlling mycobacterium infections in animals and potentially spreading to humans. The main limitation of this rule is the flaws of the tuberculin test as it can’t discriminate between animals infected with pathogenic and atypical mycobacteria. There should be permission to test the young cases below 6 months. There should be permission and support to follow up on the tuberculin-negative cases. Finally, the authorities shall adopt a new strategy for animal testing depending on highly sensitive cell-mediated immune response-based tests with molecular techniques as a confirmation to lower the flaws of the tuberculin test.

## Data Availability

The data and material are available in the manuscript.
